# Attention Network in Interpreters: The Role of Training and Experience

**DOI:** 10.3390/bs9040043

**Published:** 2019-04-23

**Authors:** Soudabeh Nour, Esli Struys, Hélène Stengers

**Affiliations:** 1 Brussels Institute for Applied Linguistics, Vrije Universiteit Brussel, 1050 Brussel, Belgium; Esli.struys@vub.be (E.S.); Helene.stengers@vub.be (H.S.); 2 Centre for Neurosciences, Vrije Universiteit Brussel, 1050 Brussel, Belgium; 3 Centre for Linguistics, Vrije Universiteit Brussel, 1050 Brussel, Belgium

**Keywords:** attention network, alerting, orienting, executive functioning, interpreting, translation, bilingualism, inhibition

## Abstract

The purpose of this study is to explore the relationship found between interpreting training and experience and the attentional network components: Alerting, orienting, and executive attention using the Attention Network Test (ANT). In the current study we tested three groups of interpreting students, translation students, and professional interpreters as specific forms of multilingual expertise. The student groups were tested longitudinally at the beginning and the end of their Master’s programme. The professional interpreters were tested only one point in time. The results showed different attention network dynamics for the interpreting students compared to the translation students regarding alertness and executive network. First, the interpreting students showed a higher conflict effect when the alert cue was presented as well as a reduced accuracy compared to translation students. Second, the interpreting training had less effect on alerting than the translation training. Finally, two student groups showed a faster response time in conflict effect than the professional interpreters. In contrast, the professional interpreters scored a higher accuracy than two-student groups specifically in an incongruent alert condition, which confirms that they used a different responding strategy.

## 1. Introduction

Attention is one of the main cognitive processes in humans. It refers to the ability of selectively focusing on relevant information while ignoring the irrelevant ones. Attention regulates different cognitive functions such as memory and language [1]. It is suggested by the behavioral studies and neuroimaging techniques that the attention system consists of three separate functional and anatomical brain areas which work together as a network. Based on the attention network theory proposed by [1,2], these three components named alerting, orienting and executive control represent different sets of attention processes [1]. Alerting as the most primitive attention network is involved in the general level of arousal and vigilance that is needed when warning or danger signals are provided. The activation of the alerting system has an effect not only on speed performance but also on accuracy. People show lower response accuracy when they need to react more rapidly to a warning signal, which is known as a speed-accuracy trade off. Orienting is involved in the direction of our attention in space or in modality based on our sensory information. It allocates the attention to particular locations or objects while trying to fixate or expect that object is there, e.g., directing our visual attention when trying to catch a ball in a game. The orienting network is more flexible and allows us to prioritize sensory information based on the information from the alerting system. The executive network is involved in our ability to sustain the attention to an object or an event and to switch between tasks. It is responsible for moment-to-moment monitoring and resolving conflicts. 

Considering the important role of attention in daily human activities, it would be beneficial if we could find some ways to promote the attention network performance. As proposed by [1], training may have possible beneficial effects on the attention network or have an impact on its underlying brain networks. Several studies have tested the effects of training on different aspects of attention network in healthy adults and patients. A study by [3], tested five-year old children using the Attention Network Test (ANT) Child, to test the effect of alerting, orienting and the executive networks in children. The experimental groups received a five-week training through the computerized exercises and were compared to the children with no training. The ERP results showed a positive effect of training on the executive network in children who received training although the behavioral results showed no difference between the two groups. Trained children activated the executive attention network faster and more efficiently than untrained children and the training effect was still present two months later without further training during that period. The positive role of training has also been seen in the executive attention after 10 weeks of Attention Process Training (APT). APT is a rehabilitation program designed to remediate attention deficits in individuals with special brain injuries. The training had a stronger influence on improving the performance of the executive attention tasks than education therapy [4]. Additionally, the training effect tested in sport domains such as Martial Arts and table tennis has shown a selective effect of training. The study by [5], showed that Martial Artists performed at a higher level in no cue conditions compare to a matched control group. In a study by [6], college table tennis athletes showed a selectively enhanced executive control of attentional network compared to non-athletes using the ANT. In addition to training, some studies tested the role of some specific long-term experience on attention network in different fields such as sport or meditation. The studies indicated that having more than 10 years of active experience in sport [7] or meditation experience had a selective positive effect on the executive network [8]. If the long time experience in some fields may help to maintain the selective aspects of attention network, how long will these effects on attentional networks last? Is it possible that these experiences have beneficial influences on these expert groups in later age, namely when the cognitive control processes start to decline due to aging? Attentional networks, like most other cognitive control functions, is affected by age. However, studies found that ageing has a selective effect on attentional networks such as a reduced alerting effect [9,10] and a reduced executive effect [9], showing an age-related slowing down of information processing rather than a general decline in the attentional networks as the orienting effect stayed intact [9].

If we look at bilingualism as a continuous spectrum which consists of different multilingual populations with a different level of proficiency, a different degree of language switching, and different language pairs, then we may predict various effects of bilingualism on cognitive control processes. Interpreters as a highly proficient bilingual group have attracted the attention of recent studies in respect to different cognitive control processes [11,12,13]. Interpreters need a high degree of language control while interpreting from a source language to a target language in a limited amount of time [14]. This time limitation and extensive degree of language switching requires a high level of attention during interpreting. But how interpreting training or interpreting experience may affect attentional control in interpreters? Does it have a selective effect on attention network components such as alerting and orienting? Following the bilingual literature studies, interpreting studies focused mainly on the executive attention in interpreters (inhibition) by using different tasks such as the Antisaccade task, Simon task and Flanker task [11,15,16,17]. The general results showed no differences between the interpreting students and the control students in the executive attention. However, testing the professional interpreters [15] found a better inhibition performance for the professional interpreters compared to translation professionals and monolinguals more specifically after the age of 34. Only four studies have tested interpreters in regard to attentional networks performance. In the first study by [18], interpreters and highly proficient bilinguals were tested using the Attention Network Test for Interaction-Vigilance (ANTI-V) which tests the attentional networks with an additional audio cue. Although no group differences were found in the three attention networks, the study showed different dynamics concerning the orienting network between two groups. The results reported a higher orienting effect for control bilinguals in the presence of a warning cue than for interpreters. The orienting effect in interpreters remained unaffected in the presence of a warning cue. It was suggested that this was the case because the level of alertness in interpreters was already high, so they did not benefit much from a warning cue. It was assumed that this different attention network interaction is due to the nature of the interpreting task. However, it was not clear whether this interaction is due to the interpreting experience or initial cognitive skills [18]. In the second study [17], two attention network components, more specifically the orienting effect and the executive effect, were tested in relation to different levels of bilingualism by comparing four groups of university students including monolinguals, unbalanced bilinguals, balanced bilinguals, and interpreter students. The results reported faster overall RTs for the three bilingual groups compared to the monolinguals. Additionally, it showed a larger orienting effect for the balanced bilinguals and the interpreters compared to the unbalanced bilinguals and the monolinguals, suggesting that the former groups benefited more from the presence of a spatial cue than the latter groups. In respect to accuracy, interpreters and balanced bilinguals performed better than the two other groups. No scores were reported for the alerting effect. In line with the two previous studies, [19] found no group differences between interpreters and a multilingual control group in terms of RTs and accuracy with no further interactions using the ANT. Finally, a longitudinal study by [20] compared three groups of students including interpreting students, translation students, and non-language students before and after their master training and showed that all three groups improved in their overall RTs, hence this training effect was present also in non-language students. No group differences or interaction effects were reported [20]. The results of these four studies on interpreters suggest that although in general there are no differences between the interpreting groups and bilinguals when testing the attention network using ANT, but different dynamics were found between networks; more specifically for the orienting network and the level of alertness in interpreters compared to controls. It is possible that these different dynamics could be related to the nature of interpreting. Further investigation is needed to shed light on the attention network dynamics in interpreters.

## 2. Materials and Methods

This study aimed to investigate the effect of both interpreting training and experience on the attention network. Considering the small amount of available literature related to the attention network in interpreters, we decided to focus on three key issues. Firstly, we were interested to look at the effect of academic interpreting training on attention network in students and replicate the only published longitudinal study by [20]. To this end, we compared two groups of interpreting and translation students longitudinally at the beginning and at the end of their one-year Master’s programme. In [18], professional interpreters showed different dynamics between alertness and orienting network from other multilingual controls, although it was not clear whether this was due to the interpreting experience or pre-existing differences. The study by [20] reported no difference between interpreter students and other control groups. The two studies, however, used a different version of the attention network tests. In [18], authors used the ANTI-V to test interpreters’ tonic and phasic alertness by using an additional audio cue and [20] used ANT which measures only phasic alertness. We should note the fact that both phasic and tonic alertness have been associated with functioning of the same neural network, but some hemispheric differences could be found in these aspects of alerting [1] and they could work independently [21]. In the present study, we first aim to replicate the only longitudinal study on the attention network in interpreter students [20], by comparing interpreting and translation students before the training and after the training using ANT. However, using ANT will not allow us to fully address the different outcomes of [18] and [20] because they used a different version of the attention task. The presence of any differences in the attention network interaction even before the start of the training between interpreting and translation students would suggest that individual differences play a role. Second, we were interested to replicate the longitudinal study by [20] in order to find out if different kinds of training have a different effect on attention network (dynamics). As the literature showed no global advantage of interpreters over balanced multilingual groups [17,18,19,20], we focus on two highly proficient bilingual student groups to better understand how specific language training might have an effect on the attention network in its global measures and dynamics. Considering differences between interpreting and translation tasks in terms of the time limitation interpreters are faced with when performing an interpreting task, we investigate if the high degree of attention to information in a short time span might have a different effect on attention network dynamics compared to translation students who do not face this time pressure. Third, we added a third group of professional interpreters with more than 20 years of active interpreting experience to explore if this kind of experience may affect their attention network performance in relation to age deterioration, which is seen most prominently in alerting and executive networks of the ageing population [9,10] and compare them with younger translation and interpreting students when they just finished their Master’s programme (post-training). 

### 2.1. Participants

Three groups of interpreting students, translation students and professional interpreters were tested using the ANT to test their attention network components: Orienting, alerting, and executive network. The two student groups were tested longitudinally while the professional interpreters were tested only once.

Thirty-eight students from the Dutch-medium Vrije Universiteit Brussel in Belgium (29 females) participated in the longitudinal experiment. All participants indicated Dutch as their dominant language (L1). Based on their Master’s programme, the population was further subdivided into two groups: The translation students and the interpreting students. Both groups’ students had obtained their bachelor’s degree in applied linguistics before entering the Master’s programme. The Master’s programme in interpreting is composed of theoretical courses and practical training (including internship) focusing on interpreting, while the Master’s programme in translation focuses more on theoretical courses and practical training in written translation. Both student groups have to choose at least two languages as their working languages. The first group consisted of 17 interpreting students (15 females) with a mean age of 22.2 years (*SD* = 1.8). The second group consisted of 21 translation students (14 females) with a mean age of 23.1 years (*SD* = 2.9). Student groups received either course credit (interpreting students) or reimbursement (translation students) for their participation in the test. The professional interpreters’ group was composed of 21 professional conference interpreters (11 females) with a mean age of 52.7 years (*SD* = 6.8) from the Directorate-General for Interpretation (DG Interpretation) of the European Commission in Brussels, who responded to the open call that was posted on the internal website of the DG Interpretation on a voluntary basis. The first language (L1) of the professional interpreters consisted of eight different languages (Dutch, French, English, German, Danish, Spanish, Romanian, and Bulgarian).

All three groups completed an adapted version of the Language Experience and Proficiency Questionnaire (LEAP-Q) [22] in Dutch or English including questions about the number of languages they spoke, their ages at onset of language acquisition for L1 and L2, self-reported interpreting or translation proficiency on a 10-point scale, the number of years of interpreting and translation experience, and the degree of exposure to the languages in the twelve months preceding the time of investigation (in percentages). The details of the participants’ background information as well as the number of participants in each test session are presented in Table 1.

### 2.2. The ANT Task

**Figure 1:**
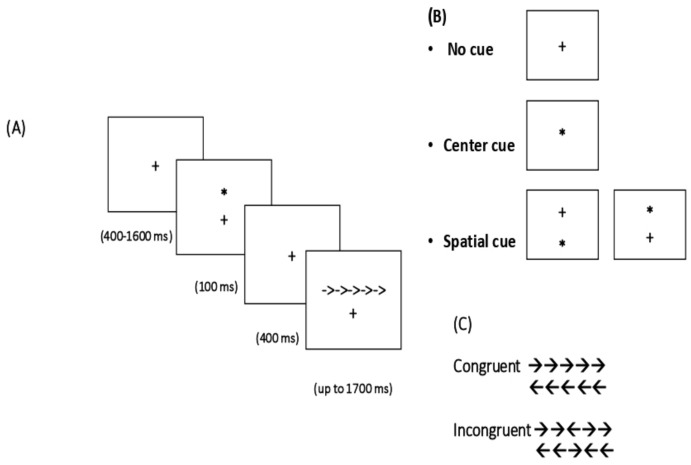
Example trial in the attention network test (ANT): The sequence of events for a trial with spatial cue for congruent trial (**A**), cue conditions (**B**), flanker type (**C**).

A shortened version of the Attention network test (ANT) with a total 144 trials was used to assess alerting, orienting and executive network. The ANT test designed by [23] is the mix of a Flanker task [24] and cue reaction time task [25]. The task goes through different steps: First a fixation cross is presented in the center of the screen (+), then for some trials a cue is presented (*) with an equal proportion of 48 trials for each cue type (24 congruent trials/ 24 incongruent trials). The cue conditions include the center cue (in the same location of the fixation cross), spatial cue (above or under the fixation cross in random) and no cue. Finally, a target stimulus is presented which is an arrow pointing to the right (→) or left (←) either above or below the fixation cross. The arrow is flanked by two additional arrows either in the same direction for congruent trials (→→→→ →) or in the opposite direction for incongruent trials (→→←→ →). The proportion of the congruent and the incongruent trails was equal (72 trials for each). The participant’s task is to respond to the direction of the CENTRAL arrow as quickly and accurately as possible. Participants should press the left mouse button if the central arrow points to the left or press the right mouse button if the central arrow points to the right. The first block was for practice and took about two minutes. The other three blocks were experimental blocks, each consisted of 48 randomized trials, and each took about five minutes. After each block there was a short break. The whole experiment took about twenty minutes (see Figure 1).

### 2.3. Procedure 

All participants were tested in the behavioral lab at the Department of Psychology and Educational Sciences of the Vrije Universiteit Brussel (VUB) in separate soundproof cabins. The participants received test instructions both orally (by the instructor) and in written form (through the monitor) before starting the test. The student groups were tested at the start of their Master’s programme and at the end of the programme, with a nine-month interval between both measurements. The professional interpreters were tested only in one point in time. The university’s guidelines regarding ethical research and scientific integrity were strictly followed. All participants gave an informed consent for participation to this experiment. The students received either course credit or reimbursement for their participation in the tests.

## 3. Results

### 3.1. Data Analysis

Firstly, the mean accuracy scores and mean response times (RT) were calculated for each subject separately. For the TR scores, the incorrect responses were excluded from further analysis and the responses that were shorter than 240 ms and longer than 1200 ms were removed to avoid outlier effects. The scores used to determine the alerting, orienting and executive control effects were calculated according to the following formulas:

- Alerting effect = (RT no cue–RT center cue)
- Orienting effect = (RT center cue–RT spatial cue)
- Executive control effect = (RT incongruent–RT congruent)

The higher alerting and orienting effects indicate the faster cue–related performance due to the presence of a warning (alerting: Cue/no cue) and place of warning (orienting: Center cue/spatial cue). However, the higher executive control effect indicates a poorer performance, as longer RTs are required for resolving the conflict. 

### 3.2. Interpreting Students vs. Translation Students

Firstly, a general mixed-model repeated measures ANOVA was performed for RTs in order to explore the interactions between the attention network components. The repeated measures model included three cue level conditions (1: Center, 2: No, 3: Spatial) and two Flanker type conditions (1: Congruent, 2: Incongruent) at two points in time (1: Pre-training, 2: Post-training) as within-subject factors. The group was defined as a between-subject factor (interpreting students and translation students). We have checked the normality distribution of the accuracy values using 1-sample Kolmogoroff-Smirnov tests for all dependent variables separately and all of these turned out to significantly deviate from the normality assumption, *p* < 0.05. As a result, different non-parametric analyses were conducted according to the study designs; the Mann-Whitney U tests were used for group measures and the Wilcoxon signed-rank for different conditions within group comparisons. Secondly, we conducted additional ANOVAs for each of the attention network components separately; the executive control effect, alerting effect and orienting effect. For these separate ANOVAs, only specific results were reported to avoid repetition of the results of the general ANOVA. (see Table 2).

The overall results for RTs showed the main effect of time, *F* (1,25) = 28.5, *p* < 0.001, *ηp2* = 0.533, flanker type, *F* (1,25) = 320.42, *p* < 0.001, *ηp2* = 0.928 and cue, *F* (1,25) = 174.12, *p* < 0.001, *ηp2* = 0.874 but no effect of the group, *F* (1,25) = 0.373, *p* > 0.05, *ηp2* = 0.015. Faster response times in post-training (M = 561.75, SD = 13.9) compared to pre-training (M = 597.9, *SD* = 12.3) were observed in two groups with a faster performance on the congruent trials compared to the incongruent trials. Significantly longer response times were found for no cue > center cue > spatial cue, respectively. Additionally, a two-way interaction was found between the time and cue, *F* (1,25) = 8.15, *p* < 0.01, *ηp2* = 0.246, which showed a less pronounced improvement for the spatial cue condition across both groups (pre-training M = 541.9, *SD* = 11.8 to post-training M = 517.05, *SD* = 13.4). Moreover, a significant two-way interaction between the flanker type and cue, *F* (1,25) = 32.84, *p* < 0.001, *ηp2* = 0.57 indicated a larger conflict effect for the center cue compared to the no cue and spatial cue conditions. A significant three-way interaction for flanker type*cue*group, *F* (1,25) = 5.34, *p* < 0.05, *ηp2* = 0.176 showed a larger conflict effect in the center cue for interpreting students.

The overall results of accuracy scores showed no effect of the group; meaning that two groups had the same overall accuracy scores in pre-training (U = 172.0, Z = −0.2, *p* > 0.05), and post-training (U = 60.5, Z = −1.5, *p* > 0.05). Further, we found a significant effect of time on the accuracy scores of incongruent trails (Z = −2.96, *p*< 0.003), more specifically the incongruent accuracy reduced in interpreting students (Z = −2.91, *p*< 0.004) in post-training (M = 93.42, *SD* = 4.53), compared to pre-training (M = 97.05, *SD* = 2.34). Additionally, the planned analysis on cue factor showed the main effect of time on the accuracy of the incongruent center cue Z = −3.37, *p*< 0.001, indicating that the incongruent accuracy reduced in the center cue condition in post-training (M = 90.27, *SD* = 7.66), compared to pre-training (M = 96.07, *SD* = 4.53).

#### 3.2.1. Conflict Effect

Following the general ANOVA, the results and the direction for the conflict effect RTs showed a main effect of the Flanker type, *F* (1,25) = 317.44, *p* < 0.001, *ηp2* = 0.927, and a main effect of time, *F* (1,25) = 30.62, *p* < 0.001, *ηp2* = 0.55, but no effect of the group, *F* (1,25) = 0.38, *p* > 0.05, *ηp2* = 0.015. There was no two-way interaction between the flanker type and group (*p* > 0.05), but there was a marginal interaction between the Flanker type and time, *F* (1,25) = 3.81, *p* < 0.06, *ηp2* = 0.132, indicating a smaller conflict effect for RTs in post-training. We did not find any three-way interaction between the flanker type* time* group (*p* > 0.05). 

The results of accuracy scores showed the main effect of time on conflict accuracy Z = −3.15, *p* < 0.002. Further analysis showed that only the scores of conflict accuracy in the interpreting group reduced significantly by time Z = −2.84, *p* < 0.004. No effect of the group was found both for pre-training (U = 174.5, Z = −0.02, *p* > 0.05), and post-training (U = 68.0, Z = −1.08, *p* > 0.05). 

#### 3.2.2. Alerting Effect

In order to measure the alerting effect and its interaction with the executive control, we performed the repeated measures ANOVA with the cue type (center cue, no cue) flanker type (congruent, incongruent) and time (pre- and post-training) as within-subject factors, and the group as a between-subject factor. 

The overall RT results followed the same direction of the general ANOVA. Two groups showed faster response times in the presence of a central cue (M = 590.13, *SD* = 12.43) than the no cue condition (M = 619.8, *SD* = 14.04). No effect of the group, *F* (1,25) = 0.34, *p* > 0.05, *ηp2* = 0.013 was found with no interaction between the cue and group (*p* > 0.05). A marginal interaction between the cue and time *F* (1,25) = 3.81, *p* < 0.06, *ηp2* = 0.132 revealed a smaller alerting effect for RTs in the post-training phase. The results also showed a significant interaction between the cue and Flanker type, *F* (1,25) = 22.81, *p* < 0.000, *ηp2* = 0.477, suggesting a larger conflict effect for the center cue than for the no cue condition. A three-way interaction between the cue*flanker type*group, *F* (1,25) = 8.8, *p* < 0.007, *ηp2* = 0.260, showed a larger conflict effect in the center cue for interpreting students. Additionally, a significant three-way interaction between the cue*time*group *F* (1,25) = 4.33, *p* < 0.05, *ηp2* = 0.148 indicated a lower degree of improvement for translation students in the center cue condition after the training. 

The accuracy results showed no effect of time on the alerting accuracy, Z = −1.60, *p*> 0.05, and no effect of the group both for pre-training (U = 146.0, Z = −1.34, *p* > 0.05), and post-training (U = 73.5, Z = −1.19, *p* > 0.05). 

#### 3.2.3. Orienting Effect

For the orienting effect and its interaction with the executive control, we performed the repeated measures ANOVA with the cue type (center cue, spatial cue), Flanker trial type (congruent, incongruent), and time (pre-training, post training) as within-subject factors, and the group as a between-subject factor. 

The overall results and direction for RTs were in line with the general ANOVA. Two groups showed a faster response time in the presence of a spatial cue (M = 529.47, *SD* = 12.16) compared to a center cue (M = 590.13, *SD* = 12.43). Moreover, we found a significant interaction between the cue type and time *F* (1,25) = 8.16, *p* < 0.009, *ηp2* = 0.246, indicating a smaller orienting effect for RTs in post-training compared to pre-training. The results showed no effect of group *F* (1,25) = 0.338, *p* > 0.05, *ηp2* = 0.013. A significant interaction between the cue and Flanker type *F* (1,25) = 32.84 *p* < 0.000, *ηp2* = 0.57, showed a higher conflict effect for the center cue than for the spatial cue. Additionally, a significant three-way interaction between the cue*flanker type*group *F* (1,25) = 5.34, *p* < 0.05, *ηp2* = 0.176 revealed a larger conflict effect in the center cue condition for interpreting students. 

The accuracy results showed no effect of time on the orienting accuracy Z = −0.06, *p* > 0.05, and no effect of the group for pre-training (U = 146.0, Z = −1.34, *p* > 0.05), and post-training (U = 87.0, Z = −0.9, *p* > 0.05).

### 3.3. Student Groups and Professional Interpreters 

In this analysis we compared the post-training scores of the two student groups in our first experiment with the scores of a group of professional interpreters. Firstly, two general repeated measure ANOVAs were performed in order to explore the interactions between attention network components. The first repeated measures ANOVA included in the model were three cue levels (1: Center, 2: No, 3: Spatial) and two Flanker types (1: Congruent, 2: Incongruent) as within-subject factors, and the group as a two-level between-subject factor (students: Post-training vs. professional interpreter). The second repeated measures ANOVA included in the model were three cue levels (1: Center, 2: No, 3: Spatial) and two Flanker types (1: Congruent, 2: Incongruent) as within-subject factors, and the group as a two-level between-subject factor (translators vs. interpreter). Dividing the group factor at two levels (age and discipline) will allow a better evaluation of the interpreting vs. translation factor. As accuracy scores were not normally distributed among participants, non-parametric Kruskal-Wallis tests are conducted to compare the three groups. Secondly, we conducted an additional planned analysis where needed to look at the executive control effect, alerting effect and orienting effect in the three groups. Table 3 showed the mean RTs and accuracy scores for alerting, orienting, and executive networks for three groups.

The RT analysis of the first ANOVA comparing students (interpreting/translation) vs. professional interpreters showed a main effect of the group, *F* (1,46) = 55.71, *p* < 0.001, *ηp2* = 0.54, with a faster performance in students (M = 560.55, *SD* = 12.2) compared to professional interpreters (M = 696.33, *SD* = 13.63). We found the main effect of the flanker type, *F* (1,46) = 764.8, *p* < 0.001, *ηp2* = 0.943, and the cue level, *F* (1,46) = 171.45, *p* < 0.001, *ηp2* = 0.788. The results showed a faster performance on congruent trials (M = 579.12, *SD* = 9.3) compared to incongruent trials (M = 673.76, *SD* = 9.20) and longer response times were found for the no cue > center cue > spatial cue, respectively. A two-way interaction was found between the flanker type and group, *F* (1,46) = 11.52, *p* < 0.001, *ηp2* = 0.200, which showed a larger conflict effect in professional interpreters *t*(46) = −3.53, *p* = 0.001 compared to students. Moreover, the two-way interaction between the flanker type and cue level, *F* (1,46) = 19.93, *p* < 0.001, *ηp2* = 0.305, indicated a larger conflict effect in the center cue condition.

The RT analysis of the second ANOVA comparing the interpreting group (students/professional) vs. translation g showed the same overall result to the first ANOVA, however contrary to the first ANOVA no interaction was found between the flanker type and group *p* > 0.05.

An additional planned analysis between the three groups showed an interaction between the alerting cue and group, *F* (2,45) = 3.69, *p* < 0.001, *ηp2* = 0.141, revealing a significant difference between professional interpreters and interpreting students for the alerting effect (*p* < 0.01); with a larger alerting effect for interpreting students, *t*(14) = 6.92, *p* = 0.001 and a smaller alerting effect for professional interpreters, *t*(20) = −2.44, *p* = 0.02. No interaction between the group and the orienting effect was found (*p* > 0.05).

The overall results of accuracy scores for the three groups showed a main effect of group (H (2) = 20.91, *p* < 0.000) due to the higher accuracy scores of professional interpreters compared to translation students (*p* < 0.002) and interpreting students (*p* < 0.000). Additional analysis showed that a better performance of professional interpreters was present only for incongruent trails (H (2) = 21.22, *p* < 0.000); regardless of the cue condition (center cue, no cue, spatial cue), for all *p* < 0.000. Planned comparison on the effect of the cue condition between the groups showed that professional interpreters gained higher accuracy scores in the center cue incongruent compared to interpreting students (*p* < 0.000) and translation students (*p* < 0.006). However, for the no cue incongruent and spatial cue incongruent professional interpreters performed only better than interpreters (*p* < 0.000), but not translation students, all *p* ns.

**Figure 2:**
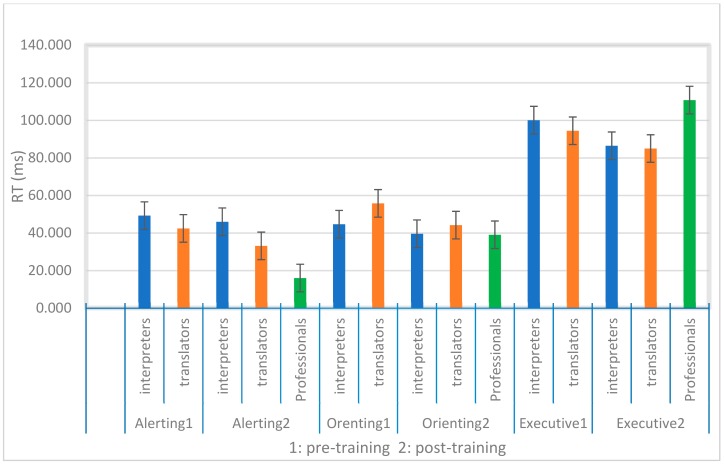
Attention network scores for response times (RTs). Error bars indicate standard error.

Additionally, the results of accuracy scores showed the main effect of the group for the conflict effect (H (2) = 20.46, *p* < 0.000), indicating higher scores for professional interpreters compared to interpreting students (U = 33.5, Z = −4.05, *p* < 0.000) and translation students (U = 39.0, Z = −3.38, *p* < 0.001). However, no main effect of the group was found for the alerting effect (H (2) = 1.87, *p* > 0.05) and orienting effect (H (2) = 2.68, *p* > 0.05) when comparing the three groups (see Table 3 and Figure 2).

## 4. Discussion

The aim of this study was to investigate the effect of interpreting training and interpreting experience on the attention network functions using the ANT. Our longitudinal study does not show any overall group differences between two student groups of interpreting and translation students before and after training. This is in line with studies by [17,18,19,20] who found no group differences comparing interpreting groups with translation students and other proficient multilingual groups. Additionally, interpreting students and translation students both showed an improvement in overall RTs scores by performing faster in post-training. In line with our study, [20] reported improvement on global RTs for interpreting students, translation students and a control student group, suggesting that this improvement is not related to language training but rather to a repetition effect. The overall accuracy scores, however, suggest a training effect as interpreting students showed a lower accuracy in post-training compared to translation students, mostly in incongruent trials. This result is also partially in line with the study by [20], in both the present study and in [20], the overall accuracy scores in translation students showed no reduction and translator students performed better than interpreter students in post-training accuracy on incongruent trials. However, this difference did not reach significance.

Although we ascertain a better performance in alerting, orienting and executive networks in post training; the decreases in response time did not follow the same pattern in both groups. The two student groups showed a smaller alerting affect at post-training, but this decrease is less prominent in the interpreting students. In other words, after the interpreting training the students stayed at almost the same level of alertness while the translation students decreased more in their alerting effect. However, this higher degree of alertness in interpreting students is not significantly higher than the translation students’ alertness. This is partially in line with the study by [18] that used ANTI-V and reported interaction between the alerting and orienting effect in interpreters compared to multilingual controls. The authors explained that presenting a tonic alerting cue was not as beneficial for the professional interpreters as for the multilingual group because the level of alertness (phasic) was already high in interpreters [18]. These results also could be explained in light of the proactive effect of the interpreter training or interpreting experience on alerting. Additionally, a neuroimaging study by [26] comparing longitudinally interpreting students and multilingual students only found an increase of cortical thickness in the attentional regions of the brain in interpreting students at post-training.

In the present study, the interpreting student group showed a larger conflict effect for the RTs while accuracy scores were lower compared to translation students in the presence of an alerting cue. In line with this finding, literature suggests a faster performance in the presence of an alerting cue compared to a no cue condition. However, this faster performance in the presence of an alerting cue condition produced a higher conflict effect [27]. In other words, the interaction between the alertness and executive effect showed that the conflict effect increases in the presence of an alerting cue [28]. This is in line with the present study’s finding while this interaction is more prominent in the interpreting students. One possible interpretation for this finding is that a high level of alertness in the interpreting students even after training causes this higher conflict effect. A high level of alertness enhances more global processing than local processing and in ANT the conflict happens at the local level [28], thus the high level of alertness in interpreter students leads to a higher conflict effect in their local conflict processing.

A comparison of the three participant groups of translation students, interpreting students and professional interpreters showed significantly faster performance in overall RTs for both student groups compared to professional interpreters. However, this students’ better performance did not apply to accuracy. Professional interpreters performed significantly better for overall accuracy scores in ANT. In order to better understand the attention network interactions in the three groups, we performed a one-way ANOVA, which showed that the three groups’ performance is on par in the orienting network, both for RTs and accuracy scores. However, as expected, the main difference was found for the conflict effect between both student groups and professional interpreters, suggesting that ageing affects the capacity for conflict resolution. This is in line with the study by [9] who found the same result comparing three groups of young, middle-aged and older adult using ANT. An additional difference was only seen in the alerting effect between interpreting students and professional interpreters. The professional interpreters were less alert than the interpreting students. However, the professional interpreters were not significantly different from the translation students in the alerting effect. As professional interpreters were compared to two student groups in post-training the outcomes should be considered as a combination of the age effect and the repetition effect. To better understand the exact effect of age and experience on alerting in the professional interpreters it is necessary to compare them with different age matched control groups (bilingual or monolingual) in future studies.

Accuracy scores followed a different pattern than the RTs. The professional interpreters obtained significantly higher total accuracy scores than the younger students. Better performances of the professional interpreters’ accuracy compared to two student groups were more prominent in the incongruent trails and more specifically for the alert cue. Additional one-way ANOVA showed that the professional interpreters’ better results applied to the executive network but not in the alerting and orienting networks. This finding showed that even if professional interpreters respond more slowly than student groups in conflict resolution, the student groups make significantly more errors. These results are confirming that younger and older adults use different strategies for responding, while younger participants rely more on speed, older adults focus more on accuracy [29,30].

In summary, the current study converges with [18] and provides more evidence that interpreters and interpreter students show different dynamics in their attentional networks compared to other multilingual groups, such as translation students. This difference was more pronounced in the alerting network both for the RTs and the accuracy. In line with the study by [31] which found better alerting performance in bilinguals compared to monolinguals; the present study goes one step further and shows that the alerting network is more robust in interpreter students even compared to translation students (as a bilingual population), but at the cost of a reduced accuracy. Although the difference in the alerting effect was not significant in the two student groups, the higher alerting effect is present in the interpreting students both in pre- and post-training which might be explained in light of individual differences in executive functions. However, due to the small number of the participants in the current study we cannot go further concerning the role of the individual differences. Additionally, alerting showed a lower decrease after interpreting training compared to translation training. Considering the lack of significant behavioral differences between both student groups on one hand and showing different attention network dynamics on the other hand, we suggest using mix research methods such as behavioral tasks, eye-tracking and EEG to better understand these attention network dynamics in interpreters preferably in a large sample size. Finally, we cannot confirm or reject that professional interpreting has a protective impact on age deterioration in attentional networks, as a higher conflict effect were found in this group compared to younger students while for the alerting effect professional interpreters showed a difference only with the interpreting students but not the translator students. Additionally, the professional interpreters performed significantly better in accuracy scores as a result of using a more efficient responding strategy. Once again, the difference was found specifically in the incongruent alert cue. Further research with the focus on the attention network dynamic and alerting system in the interpreters is suggested. We believe that the main limitation of the current study is the lack of control group for the professional interpreters. Therefore, we suggest that researchers include control groups for professional interpreters including both bilinguals and monolinguals with the same age to better understand the effect of long-term interpreting experience on the attention network. 

## Figures and Tables

**Table 1:** Of participants’ language background characteristics.

	Translation Student		Interpreting Student		Professional Interpreter	
	M	*SD*	M	*SD*	M	*SD*
**Age**	23.11	2.95	22.28	1.8	52.73	6.85
**AoA L2**	6.78	4.45	5.44	4.21	8.03	4.1
**Recent exposure L1**	50.26	18.83	47.12	14.51	40.85	17.36
**Recent exposure L2**	22.53	12.73	16.71	10.33	19.21	17.19
**TRA/INT into L1** **(self-rated proficiency)**	7.67	0.71	6.41	1.41	9.07	0.45
**TRA/INT experience** **(pre-test)**	0.00	0.00	0.00	0.00	24.60	12.15

L1: first language, L2: second language, AoA: age of acquisition, TRA: translation, INT: interpreting.

**Table 2:** Response times and accuracy scores (means, SDs) for the three groups.

	Congruent				Incongruent			
	T1		T2		T1		T2	
	M	*SD*	M	*SD*	M	*SD*	M	*SD*
**RT**								
**INT**	537.96	68.56	507.29	75.07	636.69	64.27	593.66	70.63
**TRA**	539.58	56.24	529.25	74.73	632.62	58.47	614.15	68.52
**PRO**			640.88	46.94			751.55	54.03
**ACC**								
**INT**	99.75	0.54	99.53	0.67	97.05	2.34	93.42	4.53
**TRA**	99.47	0.81	99.88	0.40	96.03	4.95	95.48	4.15
**PRO**			99.73	0.55			99.20	1.35

INT: interpreting, TRA: translation, PRO: professional interpreters, T1: pre-training, T2: post-training—RTs are reported in ms.—ACC (accuracy) scores in percentages of correct responses.

**Table 3:** Three ANT effects: Response times and accuracy scores (means, SDs) for three groups.

	Alerting				Orienting				Executive			
	T1		T2		T1		T2		T1		T2	
	M	*SD*	M	*SD*	M	*SD*	M	*SD*	M	*SD*	M	*SD*
**RT**												
**INT**	49.15	36.1	45.90	25.7	44.56	36.03	39.52	23.6	100.01	34.2	86.37	25.8
**TRA**	42.36	37.5	33.06	35.4	55.68	27.9	44.09	33.8	94.35	34.8	84.90	26.5
**PRO**			15.94	29.9			38.97	32.27			110.67	22.4
**ACC**												
**INT**	0.00	0.38	−0.27	0.46	0.00	0.37	0.07	0.26	−1.87	1.8	−4.4	3.1
**TRA**	0.25	37.5	−0.08	0.29	−0.25	0.75	−0.25	0.75	−2.1	3.6	−3.16	2.8
**PRO**			−0.09	0.44			−0.05	0.22			−0.38	1.1

INT: interpreting, TRA: translation, PRO: professional interpreters, T1: pre-training, T2: post-training—RTs are reported in ms.—ACC (accuracy) scores in percentages of correct responses.
